# Marine Ligands of the Pregnane X Receptor (PXR): An Overview

**DOI:** 10.3390/md17100554

**Published:** 2019-09-28

**Authors:** Alejandro Carazo, Přemysl Mladěnka, Petr Pávek

**Affiliations:** Department of Pharmacology and Toxicology, Faculty of Pharmacy, Charles University, Heyrovského 1203, Hradec Králové 500 05, Czech Republic

**Keywords:** natural compound, marine origin, PXR, gene regulation, CYP450, inflammation, cancer

## Abstract

Pregnane X Receptor (PXR) is a ligand-activated transcription factor which binds many structurally different molecules. The receptor is able to regulate the expression of a wide array of genes and is involved in cancer and different key physiological processes such as the metabolism of drugs/xenobiotics and endogenous compounds including lipids and carbohydrates, and inflammation. Algae, sponges, sea squirts, and other marine organisms are some of the species from which structurally new molecules have been isolated that have been subsequently identified in recent decades as ligands for PXR. The therapeutic potential of these natural compounds is promising in different areas and has recently resulted in the registration of trabectedin by the FDA as a novel antineoplastic drug. Apart from being potentially novel drugs, these compounds can also serve as models for the development of new molecules with improved activity. The aim of this review is to succinctly summarize the currently known natural molecules isolated from marine organisms with a proven ability to interact with PXR.

## 1. Introduction

Nature is an inexhaustible source of pharmaceutically useful molecules and has been exploited for this purpose since the dawn of human civilization. Both the animal and plant kingdoms have been extensively researched to provide new drugs with novel mechanisms of action or improved activity and better safety at the same time in order to find the optimal treatment of human diseases. The marine ecosystem has, for a long time already, attracted the attention of researchers for the discovery of new drugs [1]. In recent decades, research in this vast environment has been markedly intensified and has yielded a significant number of interesting molecules. The marine ecosystem, the largest in the world, provides huge biodiversity with the potential for finding many different chemicals. Cone snails, sea squirts, and marine sponges are some of the oceanic organisms from which molecules have been isolated and have served as models for the development of new therapeutic agents. Indeed, some of these compounds have been reported to possess useful pharmacologic effects. Anti-neoplastic, anti-microbial, analgesic, anti-bacterial, anti-inflammatory, anti-parasitic, anti-malarial, and neuroprotective activities are some of the therapeutic properties of compounds identified from this ecosystem. The antineoplastic trabectedin (ecteinascidin, ET-743, Yondelis^®^), an alkaloid found in the Caribbean tunicate *Ecteinascidia turbinata,* is active in the treatment of soft tissue sarcoma and other types of cancer [2,3]. Importantly, the compound has been proposed to interact with Pregnane X Receptor (PXR) as an antagonist in the presence of agonists, even though there are discrepancies regarding the molecular mechanism of the inhibition [4]. In addition, compounds isolated from marine organisms can serve as models to develop more active derivatives. For instance, the analgesic ziconotide, a synthetic analog of a conotoxin isolated from the cone snail *Conus magnus* [5], the antineoplastic cytarabine and anti-viral agent vidarabine, synthetic analogs of spongothymidine and spongouridine from the Caribbean sponge *Tectitethya crypta* [6], are clear examples of this possibility. Other promising drugs of marine origin, such as plitidepsin (anti-neoplastic), salinosporamide A (anti-neoplastic) and bryostatin-1 (anti-neoplastic), are currently undergoing clinical trials and may be available for therapy in the coming years.

**Figure 1:**
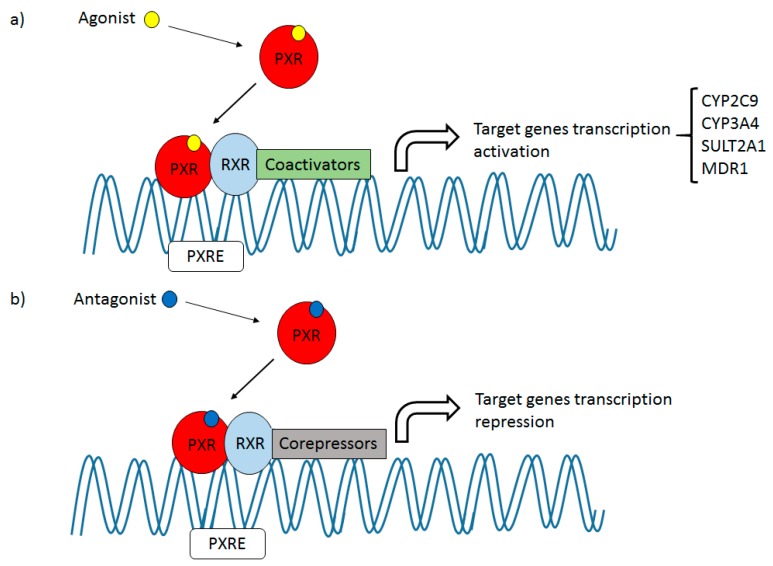
The mechanism of PXR activation with an agonist (**a**), and inhibition with an antagonist (**b**).

Pregnane X Receptor (PXR, NR1I2), first described in 1998, is a member of the nuclear receptor (NR) superfamily [7]. Upon discovery, the receptor was classified as an orphan receptor due to the lack of a known human endogenous ligand. However, the affinity between endogenous 5β-pregnane-3,20-dione and the receptor was soon established [8]. PXR was at first reported to be an important regulator of the metabolism of endogenous substances, drugs, and xenobiotics [9], but subsequent research reported a much wider array of functions in crucial physiological processes such as bile acid detoxification and elimination, glucose and lipid homeostasis, cholesterol metabolism, inflammation, bone metabolism, bilirubin clearance, oxidative stress, and cancer [10,11,12,13,14,15,16,17]. The receptor is able to play a role in these functions due to the regulation of target gene expression, through gene up-regulation or repression (Figure 1).

The utilization of new in silico methods to study the structure and characteristics of PXR and to predict the potential interactions with new ligands have significantly improved the knowledge regarding PXR [18]. Structurally, PXR possesses two key domains: a DNA binding domain (DBD) and a ligand-binding domain (LBD). DBD presents a zinc-finger structure which is the key for the identification and binding of the receptor to the DNA chain. In addition, LBD possesses the activation function-2 (AF-2), which is a crucial regulatory factor interacting directly with ligands, transcriptional co-activators, and co-repressors. Interestingly, in PXR, the LBD is relatively hydrophobic and unusually big compared to the LBDs of other NRs. This allows PXR to recognize and bind many structurally different ligands [19]. Thus, PXR is able to bind different chemical entities such as, in addition to many others, the antibiotic rifampicin, the human endogenous compound lithocholic acid, vegetal origin molecules (stilbenes and flavonoids) and the marine anti-neoplastic trabectedin [19,20].

This review summarizes the knowledge regarding natural human PXR ligands that have been isolated from marine specimens to date. Several synthetic and semi-synthetic compounds derived from compounds isolated from marine species showing activity on PXR have been developed, and some are even being used in therapy, however, these advances fall out of the scope of this review.

## 2. PXR: Drug Target

**Figure 2:**
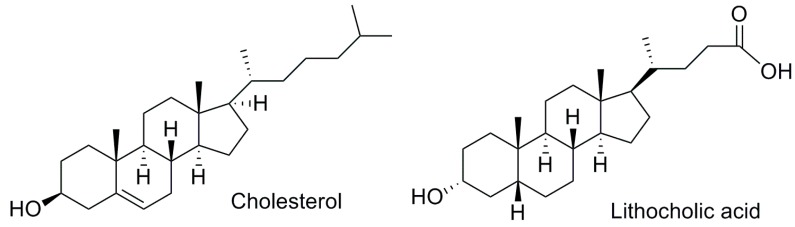
Chemical structures of cholesterol and PXR endogenous ligand lithocholic acid.

In the human organism, PXR is mainly found in the liver and the intestines, but it is also expressed in other tissues such as the colon, kidneys, breast, heart, bone marrow, and stomach [19]. In the basal state, PXR is found mainly in the cytoplasm and only after the binding of a ligand, the receptor translocates to the nucleus, where it forms a heterodimer with human retinoid X receptor (hRXR), another member of the NR superfamily (Figure 1) [19]. Depending on the nature of the attached ligand, the heterodimer complex PXR-RXR recruits co-activators or co-repressors [21,22,23]. The resulting complex is able to interact with histone acetyltransferases (HAT) and histone deacetylases (HDAC) associated with the DNA chain, allowing or preventing, respectively, the access of the transcription machinery to the genetic material and triggering or repressing genetic transcription. In addition to 5β-pregnane-3,20-dione, other endogenous human ligands have shown intense PXR activation. For instance, the secondary bile acid lithocholic acid (LCA) (Figure 2), which is toxic, showed efficient PXR activation in mice [24]. In healthy humans, LCA is detoxified through hydroxylation or conjugation pathways and is only toxic in cholestatic liver disease patients [25]. Similarly to other nuclear receptors, PXR presents species specificity. The antibiotic rifampicin is a reference human PXR (hPXR) agonist but fails to activate PXR ortholog in other organisms such as mice. Likewise, pregnenolone 16α-carbonitrile (PCN) is only able to activate mouse Pxr [9].

PXR activation plays an important role in treatment failure and undesired drug-drug interactions since the receptor regulates the expression of numerous drug transporters and phase I and II metabolizing enzymes [19]. Consequently, compounds activating PXR speed up metabolism and its activation promotes not only drug metabolism, but also transporter-mediated excretion, therefore leading to treatment failure due to decreased drug efficiency. However, not only drug-metabolizing enzymes and drug transporters are found among the target genes of PXR. The receptor also modulates key gluconeogenic enzymes (G6Pase, PEPCK1), some lipid metabolism genes (such as HMCGS2), and bile acid detoxifying enzymes (CYP3A4, SULT2A, OATP2) [10,16,26,27,28]. Nevertheless, there exists controversy regarding the role of PXR in lipid metabolism in mice models [29,30]. Additionally, several scientific studies have reported that abnormal expression or functioning of the receptor can lead to several diseases such as diabetes mellitus type II (DMII), liver diseases, hypercholesterolemia, obesity, inflammatory bowel disease (IBD), or cancer [31,32,33,34,35,36,37]. The expression of PXR in cancer has been proposed as a factor to determine the outcome of anti-cancer treatments [17]. Although PXR has been reported to participate in tissue regeneration [38], it has also been reported to be involved in the proliferation of several cancers such as colon, prostate, and breast cancers via different mechanisms ([39,40,41,42,43,44]). Importantly, PXR plays a role in the inflammatory process by antagonizing NK-κB. Indeed, PXR agonist rifampicin has been shown to possess an immunosuppressing effect in humans [13,37].

Growing evidence shows the interaction of PXR with other NRs in the regulation of different physiological processes. For instance, overlapping functions between PXR and Constitutive Androstane Receptor (CAR, NR1I3) in the regulation of drug-metabolizing enzymes and transporters, glucose and lipid homeostasis has been well documented [16,19,45,46,47]. CAR is mainly localized in the liver, brain, kidney, and lung and still lacks an endogenous high-affinity ligand [19]. The mechanisms of activation of these two closely related receptors are very similar, although CAR presents some specific characteristics. Upon the binding of a ligand, the receptor couples with retinoid X receptor (RXR) and recruits the rest of the necessary transcription machinery. However, CAR can also be activated in absence of a ligand [19,48]. The cross-talk between these receptors is evidenced as CAR and PXR have been proven to share some common activators, such as phenobarbital and 5β-pregnane-3,20-dione [8,49]. Interestingly, several compounds have shown inverse activity on these receptors. For instance, the antifungal drug clotrimazole is a PXR activator, yet behaves as a CAR antagonist [49].

Farnesoid X Receptor (FXR, NR1H4) regulates bile acid homeostasis and is mainly found in the liver, but also in the kidneys, intestines and adrenal glands. The main ligands of the receptor are endogenous bile acids, and chenodeoxycholic acid (CDCA) is its reference ligand [50]. Similar to PXR and CAR, FXR also dimerizes with RXR upon the presence of a ligand and triggers or represses genetic transcription. Importantly, in addition to FXR, PXR has been shown to possess a high affinity towards bile acids. PXR is able to regulate not only bile acid homeostasis but also lipid and cholesterol synthesis, metabolism and transport [50,51,52,53,54].

Thus, cross-talk of PXR with other members of the NR superfamily should be considered when considering PXR as a drug target.

## 3. Marine PXR Ligands

Multiple compounds isolated from marine organisms have been studied as potential therapeutic tools and used as models for the development of semi-synthetic and synthetic derivatives. In recent decades, the relevance of NRs as drug targets has been increasing, and the research for new marine ligands has also been intensified. These efforts have yielded an important number of new compounds able to interact with these therapeutic targets.

**Figure 3:**
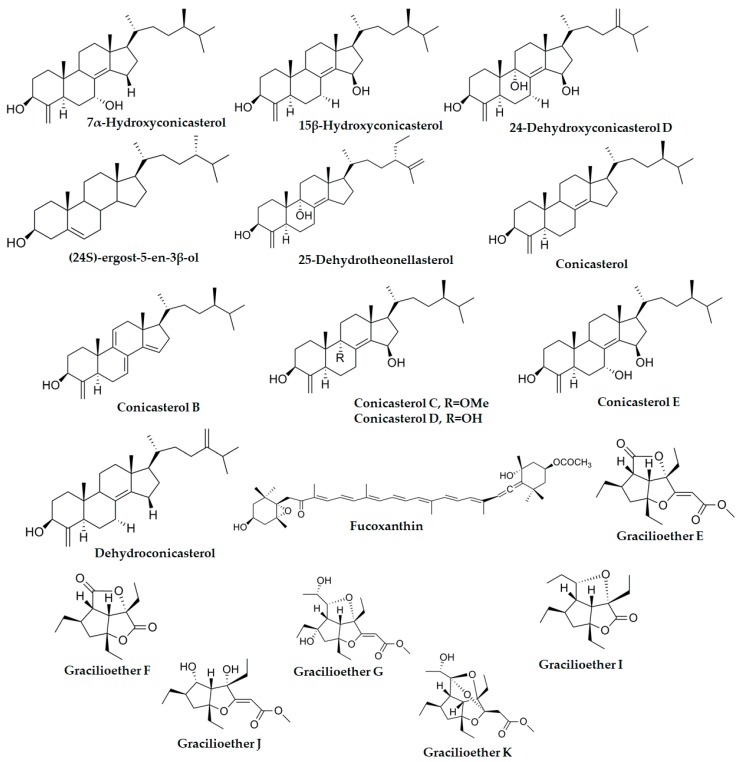
Chemical structures of marine origin molecules with known PXR activity.

**Figure 4:**
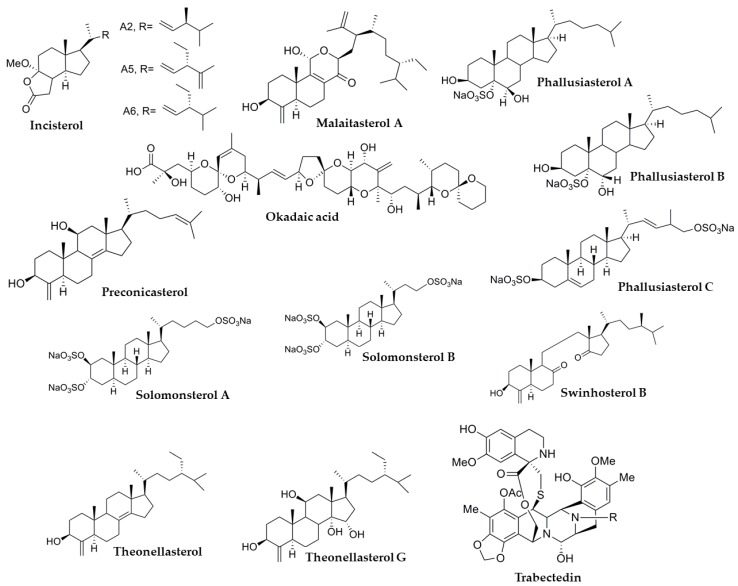
Additional chemical structures of the marine origin molecules with known PXR activity.

Marine sponges, algae and tunicates are some of the oceanic specimens with a huge potential to synthesize useful molecules. In particular, sponges have provided a significant number of molecules tested on several NRs, including PXR. Also, other marine organisms can produce interesting molecules and it is reasonable to assume that many novel molecules will be discovered in the near future. In this section, natural molecules with reported activity on human PXR are reported (Table 1). Their chemical structures are shown in Figure 3 and Figure 4.

### 3.1. Theonella sp.

*Theonella* sp. is a genus of marine sponges, first described in 1868, originating from the west Pacific and Indian Oceans. Sponges from this genus have been reported to provide a significant number of compounds that are able to interact with PXR. Importantly, all the active compounds isolated from these species have a steroidal structure and most of them have been isolated from two species of the *Theonella* family, namely *T. swinhoei* and *T. conica*.

#### 3.1.1. Solomonsterols

Solomonsterols A (SolA) and B (SolB) are two of the active compounds isolated from the sponge *T. swinhoei.* Both compounds, which differ only in the length of their side chains (Figure 4), have been reported to activate PXR in transfected HepG2 cells and enhanced the expression of PXR target genes such as efflux transporter P-glycoprotein/MDR1 and phase I metabolizing enzyme CYP3A4 [59,60,61]. Since they are able to activate PXR, it is understandable to suppose that these compounds may have an anti-inflammatory effect. Thus, these compounds have been tested in animal models and mouse cell lines to study this potential activity. Festa et al. showed that both compounds, SolA and SolB, were able to revert the inflammation triggered by LPS and IL-8, but not due to TNF-α in RAW 164.7 mouse macrophage cell line. This effect was confirmed in mice with induced rheumatoid arthritis (RA), where SolA inhibited the expression of the inflammatory cytokines TNFα and IFNγ [61]. In addition, docking assays confirmed the interaction of solomonsterol A with the PXR-LBD [62].

#### 3.1.2. Theonellasterols

A total of 9 structurally closely related steroids were isolated from *Theonella swinhoei*: theonellasterols B-H, J, and theonellasterol (Figure 4). The PXR activity of these compounds was tested in vitro on transfected HepG2 cells [55,63,64]. All compounds were reported to activate the receptor, however, expression of the PXR target genes MDR1 and SULT2A1 was enhanced only by theonellasterol G in HepG2 cells. In addition, a dual activity on PXR and FXR was observed. Theonellasterol G was able to activate both PXR and FXR [64]. In addition, the authors also reported enhancement of the target expression genes of PXR (SULT2A1, MDR1) and FXR (BSEP, OSTα) involved in drug and bile acid metabolism and transport.

Theonellasterol weakly activated PXR in HepG2 cells and showed no activity on FXR [65]. However, the compound was able to revert the chenodeoxycholic acid activation of FXR as well as the expression of FXR target genes (OSTα, BSEP, and SHP) in HepG2 cells.

Lastly, theonellasterols B, D, E, and F showed an intense agonistic effect due to binding to the ligand-binding domain of PXR in docking assays [64].

#### 3.1.3. Conicasterols

Conicasterols B, C, D, E, G-K, and conicasterol (Figure 3), are other steroidal compounds isolated from *T. swinhoei* with a significant PXR activating effect in transfected HepG2 cells with luciferase responsive constructs [63,64,66].

Conicasterol, found both in *T. swinhoei* and *T. conica*, was reported to intensely activate PXR in HepG2 transfected cells [63]. Interestingly, conicasterol was also able to repress the effect of PXR agonist rifaximin. The affinity of the compounds to PXR-LBD was studied by docking assays, and conicasterol D showed a weak agonistic effect in this method [64].

Conicasterol E was able not only to activate PXR but also FXR [66]. These effects were subsequently proved in HepG2 cells where significant enhancement of the expression of the PXR target gene CYP3A4 and the FXR target genes OSTalpha, BSEP, CYP7A1, SHP was observed. Moreover, conicasterol J also showed dual activity: it is able to activate PXR and to antagonize FXR, and this was also observed in the presence of FXR agonist chenodeoxycholic acid, similar to the behavior of theonellasterol G [63].

#### 3.1.4. Swinhosterol B

De Marino et al. (2012) reported that swinhosterol B (Figure 4), another sterol isolated from *T. swinhoei*, behaved not only as a PXR agonist but also as an FXR repressor in HepG2 transfected cells with a luciferase reporter assay [63]. The compound was reported to activate PXR to a similar degree as the reference activator rifampicin at a concentration of 10 µM. Furthermore, swinhosterol B showed anti-inflammatory activity, being able to repress the expression of TNFα, IL-1β, and IL-6 triggered by LPS in mice expressing human PXR [63].

Additionally, the authors reported that the expression of the FXR target genes OSTα, SHP and BSEP was repressed by swinshosterol B both alone and in combination with chenodeoxycholic acid in HepG2 cells.

#### 3.1.5. Malaitasterol A

Malaitasterol A is another steroidal compound isolated from the marine sponge *Theonella swinhoei* (Figure 4). This sterol was documented to activate not only PXR in transfected HepG2 cells but also PXR target genes. The activation of these phase I and II metabolic genes and drug transporters such as CYP3A4, MDR1, and SULT2A1, was reported to be similar to the reference control rifaximin [67]. Moreover, and similarly to other sterols isolated from *Theonella* sp., in addition to the PXR agonistic activities, malaitasterol A behaved as an FXR antagonist in HepG2 cells.

#### 3.1.6. Preconicasterol

Preconicasterol is another of the compounds isolated from *Theonella swinhoei* (Figure 4). The compound was reported to intensely activate PXR in transfected HepG2 cells [55]. Activation by preconicasterol showed to be dose-dependent (EC_50_ = 21 µM). In addition, after treatment with preconicasterol, PXR target gene (CYP3A4, MDR1, and MDR3) mRNA expression was enhanced in HepG2 cells. However, the expression of functional PXR in this cell line is minor, which may open a discussion over these results.

#### 3.1.7. Other Compounds from *Theonella* sp.

Several other compounds from *Theonella* sp. have been isolated and tested for PXR activity. Among them are dehydroconicasterol, 24-dehydroconicasterol D, 25-dehydrotheonellasterol, 7α-hydroxyconicasterol, and 15β-hydroxyconicasterols (Figure 3). All of these compounds were reported to activate PXR in transfected HepG2 cells using a reporter assay [55,63].

In addition, 7α-hydroxyconicasterol and 15β-hydroxyconicasterols were also tested on FXR. However, the compounds had no significant activity on this receptor in HepG2 cells [63].

### 3.2. Phallusia fumigata

*Phallusia fumigata* is a marine squirt mainly found in the Atlantic and Pacific Oceans, but can also be encountered in the Mediterranean Sea. Research efforts have identified three active sterols from this species, phallusiasterols A, B and C (Figure 4).

In PXR transfected HepG2 cells, phallusiasterol A induced intense activation, but phallusiasterols B and C did not show any significant activation of the receptor [68,69]. In addition, phallusiasterol A was able to enhance the mRNA expression of the PXR target genes CYP3A4 and MDR1 in HepG2 cells [68].

### 3.3. Plakortis lita

*Plakortis lita* is a sponge found in the western Pacific Ocean. Several compounds with an endoperoxide structure were isolated from the sponge and tested for activity on PXR: incisterols A2, A5, and A6 (Figure 4). Chianese et al. studied the effect of these compounds on PXR transfected HepG2 cells. Incisterols A5 and A6 activated PXR to a similar extent as the positive control rifaximin, however, incisterol A2 showed a weaker activation [70]. In addition, enhanced expression of the PXR target genes CYP3A4 and ABCB1/MDR1 was reported only after A5 and A6 treatment in the same cell line.

### 3.4. Plakinastrella mamillaris

*Plakinastella mamillaris* is a sponge found in the eastern Indian Ocean. Several PXR active compounds called gracilioethers have been isolated from this sponge. In total, six polyketides were isolated from the organism: gracilioethers E-G and I-K (Figure 3). All gracilioethers, except for gracilioethers F and K, were able to activate PXR in HepG2 cells to a similar degree as rifaximin [71]. The compounds were also tested on FXR, but no activation was observed. The authors also provided docking studies showing the interaction of the compounds with the ligand-binding domain of PXR to support their findings.

### 3.5. Ecteinascidia turbinata

The marine squirt *Ecteinascidia turbinata* is found in the north of the Atlantic Ocean and the Mediterranean Sea and it is the source of the widely known alkaloid trabectedin (ecteinascidin-743, ET-743, Yondelis^®^, Figure 4). Trabectedin is used in the treatment of soft tissue sarcoma, breast, ovarian and other cancers [2,72,73,74,75], and it is currently under trial against other kinds of cancer [76]. The major drawback seems to be its hepatotoxicity observed in clinical trials.

In an elegant study, Ekins et al. predicted the interaction of trabectedin with PXR using computational methods [77]. This hypothesis was confirmed within in vitro experiments, where the PXR antagonist activity of the compound was clearly established. The drug behaves like a PXR antagonist and is able to abolish the receptor activation after treatment with well-established activators (paclitaxel or SR12813) with an IC_50_ of 3 nM in CV-1 cells [34]. In addition, independent works have reported a repressive effect of trabectedin on PXR target genes such as MDR1 in human colon carcinoma cell line SW620 and CYP3A23 (the major CYP3A isoform in rats) in rat hepatocytes. These results suggest this effect is a consequence of PXR antagonism [78,79].

### 3.6. Sinularia kavarattiensis

Sinularia is a genus of soft coral present in the Indian Ocean. In recent work, five sterols isolated from *Sinularia kavarattiensis* were tested for potential activity in PXR (Figure 3) [58]. Among these isolated natural compounds, only (24S)-ergost-5-en-3β-ol) showed significant activation of PXR. This activation was dose dependent and EC_50_ was stablished at 2.3 µM. In addition, this sterol was able to activate the expression of CYP3A4 in HepG2 cells [58].

### 3.7. Undaria pinnafitida

Fucoxanthin is a carotenoid found in the algae *Undaria pinnafitida*, also known as wakame. This alga is highly invasive and thus can be found in the Atlantic, Pacific, and Indian Oceans. The plant has been used in Chinese medicine as a “blood purifier” and a hair growth stimulator, as well as a menstrual stabilizer. The plant is a source of calcium and rich in eicosapentaenoic acid (EPA), ω-3 fatty acids and carotenoid fucoxanthin (Figure 3). Importantly, wakame is part of the traditional diet in Japan and China. Fucoxanthin has been reported to have anti-cancer [80,81,82], anti-inflammatory [83], and hypolipidemic properties [84].

Interestingly, in a recent study, fucoxanthin was reported to revert the expression of PXR and to decrease the expression of its target genes. In HepG2 cells, fucoxanthin was able to prevent the transactivation of PXR in the presence of the positive ligand rifampin and to decrease the protein and mRNA expression of CYP3A4 and ABCB1/MDR1 [85]. The authors also reported an inhibitory effect of fucoxanthin in the recruitment of coactivator SRC-1. In light of these results, the authors proposed an anti-drug resistance activity for fucoxanthin through the inhibition of metabolizing enzymes and drug transporter expression.

### 3.8. Dinophysis sp.

Dynoflagellates (*Dinophysis* sp.) are found in water habitats, mainly as a part of marine plankton. These organisms produce a polyketide toxin called okadaic acid (Figure 4), which is accumulated in marine sponges, bivalve mollusks, fish and shellfish. Its mechanism of action is based on the inhibition of phosphatases 1 and 2A. This causes gastrointestinal disorders after the consumption of marine products contaminated by the toxin [86]. Furthermore, okadaic acid has neurotoxic and immunotoxic effects, which hinders its potentially useful effects on Alzheimer disease, diabetes, and AIDS [87,88].

Regarding its activity on PXR, two recent works have reported a weak PXR activation in transfected HepG2 cells by okadaic acid [56,57]. Both research papers reported a dose-dependent activation of the receptor by the toxin, however, with some difference between the EC_50_ values. Fidler et al. reported an EC_50_ of 7.2 nM, whereas Ferron et al. reported 33.85 nM. This difference is probably due to the different cell types used in each study. In addition, okadaic acid was shown to weakly enhance the activation of the PXR target gene CYP3A4 in HepaRG cells [57].

### 3.9. Other Compounds That Resemble PXR Activation Effects but do Not Directly Activate PXR

There are other natural compounds isolated from marine species which have been reported to activate PXR target genes or to have activities similar to PXR activation but have failed to directly activate the receptor.

**Figure 5:**
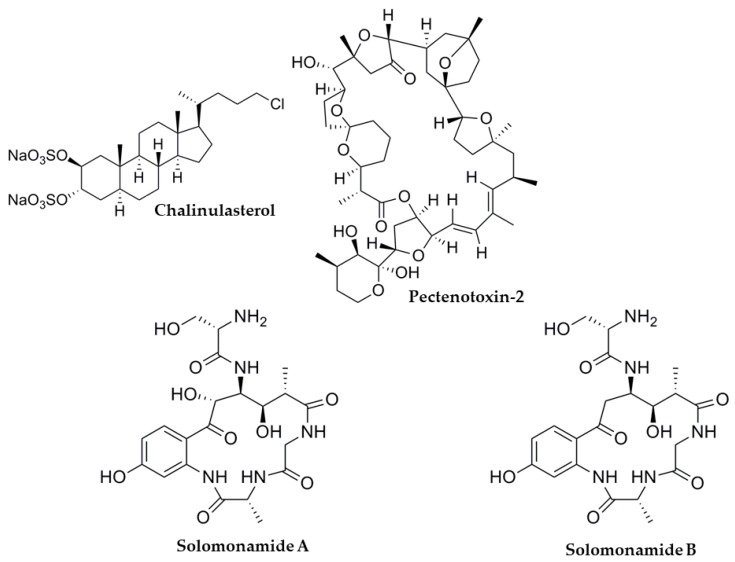
Compounds activating PXR target genes but unable to directly activate the receptor.

Chalinulasterol is another marine-origin steroid, isolated from the Caribbean sponge *Chalinula molitba*. The compound showed no activity in PXR transfected HepG2 cells and did not enhance the expression of CYP3A4 in HepG2 cells [60]. This finding is very interesting since the only structural difference between solomonsterol A and B, known PXR activators, is the replacement of the polar sulfate group by a chlorine atom in the side chain of the molecule (Figure 5).

Other compounds isolated from *T. swinhoei* that have not been tested for PXR activity include solomonamides A and B (Figure 5) [89]. However, authors have reported that solomonamide A has anti-inflammatory properties in mice [89], which may indicate a potential PXR agonistic activity.

Pectenotoxin-2 (PTX-2) (Figure 5) is a toxic macrolactone isolated from the alga *Dinophysis fortii* [90] with potential anti-cancer activity [91,92]. A work by Fidler et al. reported that this toxin showed no significant activation of the PXR [56]. However, a different work reported no direct PXR activation, but weak enhancement in PXR target gene (CYP3A4, CYP2C9, CYP2C19) expression in HepaRG cells [57]. In addition, pectenotoxin-2 and okadaic acid share reasonably similar structures, which may explain that both molecules cause diarrheic shellfish poisoning. However, this is probably the reason behind the observed difference in PXR activity.

Interestingly, two sterols (scallaristerol and callysterol) isolated from marine sponges from the Red Sea (*Scalarispongia acabensis* and *Callyspongia siphonella*, respectively), were reported to have anti-inflammatory properties [93], but no effect on PXR has been studied yet to our knowledge. Once again, it is reasonable to hypothesize these compounds may exert their activity through the PXR-activation pathway.

Lastly, recent work has reported five new compounds isolated from the marine sponge genus *Myrmekioderma* from Hawaii, but only a very mild PXR activation was shown only for one of these compounds [94].

## 4. Therapeutic Use and Potential Clinical Applications

**Figure 6:**
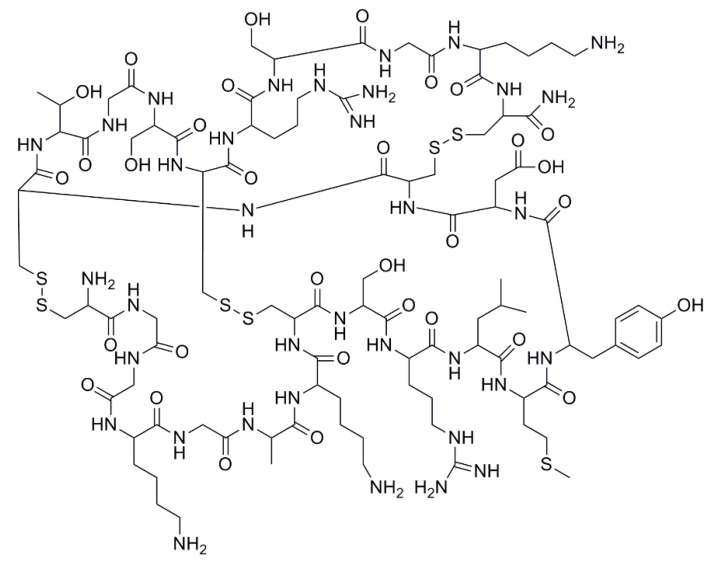
Chemical structure of ziconotide.

After the identification and isolation of a new molecule from an organism, there is still a long way to go until the potential new drug reaches its first pre-clinical steps, then clinical trials and finally, approval for use in human therapy. The advances in research, isolation techniques, structure elucidation, and experimental procedures have improved the process of identifying new molecules. Several marine-origin molecules are already being used in therapy, such as the anti-cancer trabectedin and the analgesic ziconotide (Figure 6) [2,3,5,6]. Apart from these compounds, many molecules with a marine origin have shown potential therapeutic activities, especially in cancer. For a comprehensive review, see Calcabrini et al. 2017 [95].

Most of the molecules reported in this review have been isolated and identified quite recently and thus, their potential therapeutic utility is yet to be fully established. However, for some compounds, potential therapeutic indications have already been described. For instance, okadaic acid has been reported to be a potential tool in diabetes, Alzheimer disease, and AIDS, although it has proven to possess high toxicity [87,88]. Interestingly, solomonsterols A and B and swinhosterol B were reported to repress inflammatory cytokines, probably through a PXR-mediated mechanism [59,63]. However, the molecular mechanisms for most of these molecules and many others remain to be fully clarified.

Interaction with PXR has been reported for a wide variety of compounds from animal, vegetable, synthetic, and semisynthetic origins. The first known agonist for hPXR was a human endogenous hormone: 5β-pregnane-3,20-dione [8]. Since then, many other compounds have been reported to interact directly with the receptor. The antibiotic rifampicin is considered to be the standard hPXR agonist [96], whereas trabectedin, ketoconazole, sulforaphane, and metformin are known PXR antagonists [34,97,98]. However, even though many of these drugs have been reported to intensely activate PXR, this activation is significantly lower than that of rifampicin in all cases, which could present a problem in their potential application in therapy. The intense interaction between rifampicin and the ligand-binding domain of PXR is well documented. Although the molecule is quite big, docking studies reported that it is able to directly interact with key residues in that domain forming important hydrophobic contacts or hydrogen bonds [19]. Most of the compounds reported in this review share a common chemical structure that is much smaller than rifampicin and thus, they fail to establish so many interactions with PXR. This fact may explain the lower intensity of the activator effect. However, these steroidal structures are closely related to the endogenous bile acid lithocholic acid, which is another high-affinity ligand of PXR [24]. Interestingly, steroids of both *trans* A/B rings conformation, such as lithocholic acid, as well as *cis* conformation, like most of the marine PXR ligands here reported, are able to activate hPXR. Therefore, the different effect of rifampicin and lithocholic acid in relation to the molecules reported in this review must be influenced not only due to the direct interaction of the molecule with amino acid residues in the ligand-binding domain of PXP but also with other molecular factors.

There is always space to improve the current palette of clinically used drugs. In this context, the marine environment represents a vast source of new potential compounds which may provide novel active compounds. In addition, some of these compounds may also serve as a model for the further development of new chemical entities (NCE) [99]. From the therapeutic point of view, not only PXR activation but also inhibition of its effect can result in positive outcomes with potential use in human medicine since it has been reported to be involved in liver conditions, inflammatory processes, DMII, and cancer promotion [13,31,33,34,36,37]. The therapeutic effect of PXR is controversial. Mostly, PXR activation is undesired, since it has been reported to be involved in xenobiotic metabolism, obesity, cholesterol metabolism, atherosclerosis, cardiovascular conditions, and cell proliferation [9,100,101]. However, PXR activators can potentially be used in several indications. Rifampicin is a macrolide antibiotic widely used in the treatment of tuberculosis and other bacterial infections. In addition, and only marginally, it is also used in the treatment of cholestatic pruritus [102]. Since rifampicin is a strong PXR activator, it is possible to suppose that this effect may be mediated through the promotion of bile acid excretion into the bile via the upregulation of several metabolizing enzymes such as CYP7A1 and CYP27A1 [103]. However, further research is needed to asses if other PXR agonists may also be useful in the treatment of other indications. On the other hand, PXR antagonism seems to have more beneficial effects. A PXR antagonist may decrease drug failure or resistance to pharmacological treatment [104]. At present, some natural molecules with a marine origin are already known to antagonize PXR and are currently being used as anti-cancer agents, such as trabectedin and fucoxanthin [2,3,34,36,85]. Also, several other compounds with a marine origin have been reported to have activities not only in cancer but also in inflammatory processes [105]. Interestingly, a recent study has reported that PXR can be synergistically activated by several molecules at the same time [106]. This fact opens new possibilities in the study of the mechanistic interactions of molecules that interact with the receptor. Although the role of PXR in some of the previously reported conditions is known to some extent, there are other processes in which the function of the receptor is yet unknown. It is our opinion that further research, especially clinical studies, will help to decipher the relevance of the receptor in the pathophysiological processes in which it is involved.

In order to determine the mechanism of action and potential activities of these compounds, it is necessary to test the molecules described in this work on a wider range of molecular targets, for instance, with other fellow NRs. For some of the compounds reported in this review, assays have been performed in the fellow nuclear receptor FXR. Interestingly, some of the molecules showed a dual activity. For example, theonellasterol showed a PXR agonistic activity, yet it was able to antagonize FXR in transient transfected HepG2 cells [65]. In addition, conicasterol H, J, conicasterol, swinhosterol B and theonellasterol G showed intense repression of the activation of the FXR reference positive control CDCA in transfected HepG2 cells, however, they all behaved as PXR agonists [63,64]. Lastly, other compounds such as conicasterol E and theonellasterol G showed agonistic effect both in PXR and FXR [64,66]. However, the potential activities of these compounds on other biological targets remain to be tested and established.

Ziconotide and auristatin, other compounds derived from a marine origin, are already being used and, to the best of our knowledge, have not been assayed on PXR. Ziconotide, a synthetic analog of a conotoxin isolated from the marine snail *Conus magnus*, is used as an analgesic in cases of chronic intense pain [107]. Auristatin, a monomethyl derived from dolastatin 10, a cytotoxic peptide isolated from the cyanobasteria *Caldora penicillata*, is used in combination with a tumor-specific antibody for the treatment of Hodgkin’s lymphoma and other types of cancer [108]. The authors believe it would be interesting to test these compounds on PXR and study their effects.

## 5. Concluding Remarks

The marine environment accounts for approximately 70% of the surface of the planet. Understandably, the number of organisms living in this ecosystem is a vast potential source of novel molecules. In recent decades, growing efforts have been focusing on this environment. Although research has so far provided an important number of promising molecules from the marine ecosystem, at present, only a small group of marine-origin drugs is currently being used in therapy. Among them, trabectedin is probably the best-known drug and is used as an antineoplastic agent in several types of cancer (2,69–72). Since several of these marine-origin compounds are still undergoing clinical trials, it is reasonable to believe that some of these molecules may be useful in the future for the treatment of different conditions.

The importance of NRs as therapeutic targets is rapidly increasing and with the development of personalized medicine, their importance will surely grow in the coming years. Thus, the discovery of new ligands can be key for the development of new tools and will help to achieve a better understanding of the functioning of these receptors. Most of the compounds reported here have been tested only on FXR in addition to PXR. In these studies, the data showed dual activity of some of these compounds [63,65,71]. These two receptors, and other members of the NR superfamily, are known to be able to regulate the expression of many genes involved in different physiological processes and functions, and the cross-talk between them has been well documented [19,51,109]. Despite most of the experiments being performed in human hepatic cell lines, we believe that gene expression studies would provide additional information if performed on other relevant cell lines. For instance, HepaRG cells and the gold standard human hepatocytes are well-established models for the study of gene expression in the liver [110].

Given the extent of this ecosystem, more interesting compounds with novel structures and useful properties remain to be discovered. The possibility of finding a groundbreaking molecule for the treatment of some condition in this environment is a plausible possibility in the near future. However, the availability and accessibility of some of these species is a challenge in this research.

## Figures and Tables

**Table 1:** Natural molecules from marine organisms with reported activity on Pregnane X Receptor (PXR).

Species	Compound	Type of Molecule	PXR Effect	EC_50_/IC_50_
*Theonella swinhoei*	7α-Hydroxiconicasterol	Steroidal	Agonist	NT
	15β-Hydroxyconicasterol	Steroidal	Agonist	NT
	24-Dehydroconicasterol D	Steroidal	Agonist	NT
	25-Dehydrotheonellasterol	Steroidal	Agonist	NT
	Conicasterol B, C, D, E	Steroidal	Agonist	NT
	Dehydroconicasterol	Steroidal	Agonist	NT
	Malaitasterol A	Steroidal	Agonist	NT
	Preconicasterol	Steroidal	Agonist	EC_50_ = 21 µM [55]
	Solomonsterol A, B	Steroidal	Agonist	NT
	Swinhosterol B	Steroidal	Agonist	NT
	Theonellasterol G	Steroidal	Agonist	NT
	Theonellasterol	Steroidal	Agonist	NT
*Theonella conica*	Conicasterol	Steroidal	Agonist	NT
*Phallusia fumigata*	Phallusiasterol A-C	Steroidal	Agonist	NT
*Plakinastrella mamillaris*	Gracilioethers E-G,I-K	Polyketide	Agonist	NT
*Plakortis* cfr. *lita*	Incisterols A2, A5, A6	Steroidal	Agonist	NT
*Dinophysis sp.*	Okadaic acid	Polyketide	Agonist	EC_50_ = 7.2 nM [56] EC_50_ = 33.9 nM [57]
*Ecteinascidia turbinata*	Trabectedin (ET-743)	Alkaloid	Antagonist	IC_50_ = 3 nM [34]
*Undaria pinnafitida*	Fucoxanthin	Carotenoid	Antagonist	NT
*Sinularia karavatensis*	(24S)-ergost-5-en-3β-ol	Sterol	Agonist	EC_50_ = 2.3 µM [58]

N.T.: not tested.
